# Implication of ERBB2 as a Predictive Tool for Survival in Patients with Pancreatic Cancer in Histological Studies

**DOI:** 10.3390/curroncol29040198

**Published:** 2022-03-30

**Authors:** Miguel A. Ortega, Leonel Pekarek, Oscar Fraile-Martinez, Cielo Garcia-Montero, Miguel A. Saez, Angel Asúnsolo, Miguel A. Alvarez-Mon, Jorge Monserrat, Lidia Ruiz-Llorente, Natalio García-Honduvilla, Agustin Albillos, Julia Buján, Melchor Alvarez-Mon, Luis G. Guijarro

**Affiliations:** 1 Department of Medicine and Medical Specialities, Faculty of Medicine and Health Sciences, University of Alcalá, 28801 Alcala de Henares, Spain; leonel.pekarek@edu.uah.es (L.P.); oscar.fraile@edu.uah.es (O.F.-M.); cielo.garcia@edu.uah.es (C.G.-M.); msaega1@oc.mde.es (M.A.S.); angel.asunsolo@uah.es (A.A.); maalvarezdemon@icloud.com (M.A.A.-M.); jorge.monserrat@uah.es (J.M.); natalio.garcia@uah.es (N.G.-H.); agustin.albillos@uah.es (A.A.); mjulia.bujan@uah.es (J.B.); mademons@gmail.com (M.A.-M.); 2 Ramón y Cajal Institute of Sanitary Research (IRYCIS), 28034 Madrid, Spain; lidia.ruizl@uah.es (L.R.-L.); luis.gonzalez@uah.es (L.G.G.); 3 Cancer Registry and Pathology Department, Principe de Asturias University Hospital, 28806 Alcala de Henares, Spain; 4 Oncology Service, Guadalajara University Hospital, 19002 Guadalajara, Spain; 5 Pathological Anatomy Service, Central University Hospital of Defence-UAH Madrid, 28801 Alcala de Henares, Spain; 6 Department of Surgery, Medical and Social Sciences, Faculty of Medicine and Health Sciences, University of Alcalá, 28801 Alcala de Henares, Spain; 7 Unit of Biochemistry and Molecular Biology, Department of System Biology, University of Alcalá, 28801 Alcala de Henares, Spain; 8 Department of Gastroenterology and Hepatology, Ramón y Cajal University Hospital, University of Alcalá, Ramón y Cajal Institute for Health Research, 28034 Madrid, Spain; 9 Biomedical Research Networking Center of Hepatic and Digestive Diseases (CIBEREHD), Institute of Health Carlos III, 28034 Madrid, Spain; 10 Immune System Diseases-Rheumatology, Oncology Service an Internal Medicine, University Hospital Príncipe de Asturias, 28806 Alcala de Henares, Spain

**Keywords:** pancreatic cancer, EGFR, Cyclin D1, CDK4, ErbB2, prognostic factors

## Abstract

Pancreatic cancer will be positioned by the year 2030 as the second cause of oncological death after lung cancer. The pathophysiology of the most common variety, which involves the adenocarcinoma of the pancreas, represents one of the main challenges for current oncology to explain its tumorigenesis and create a targeted treatment. The tumor microenvironment, metastatic capacity, and lack of early diagnosis lead patients to present advanced stages at the time of diagnosis. Despite numerous efforts, little progress has been made in clinical outcomes and with respect to the improved survival of these patients. For this reason, in recent years, numerous diagnostic tests, treatments, and possible approaches in the fields of radiotherapy, chemotherapy, immunotherapy, and surgery have been developed to find a combination of methods that improves life expectancy in patients diagnosed with this disease. On the other hand, the scientific community has made numerous advances in the molecular bases of pancreatic cancer since several oncogenetic pathways have been described and the markers expressed by the tumor have proven to be useful in the prognosis of pancreatic adenocarcinoma. These molecular alterations allow the study of possible therapeutic targets that improve the prognosis of these patients, but even numerous tumor cell-individual interactions must be explained to understand the underlying pathophysiology causing the high mortality. Therefore, the purpose of our study is to examine the expression of markers such as EGFR, Cyclin D1, andCDK4 in order to find a relationship with the possible long-term prognostic factors of patients affected by pancreatic ductal adenocarcinoma. Our results show that there is a prognostic role for ErbB2, EGFR, beta catenin, cyclin D1, and CDK4. Of these, we highlight the clinical importance of ErbB2 in the survival rates of patients who overexpress this component.

## 1. Introduction

Pancreatic cancer is a potentially lethal malignancy representing the seventh cause of cancer-related deaths worldwide [1]. Pancreatic ductal adenocarcinoma is the most common type of pancreatic cancer, accounting for around 90% of total cases [2]. The incidence of this cancer is increasing worldwide, with little or no improvements in overall survival [3]. Indeed, it is expected that, by 2030, pancreatic cancer will become the second cause of cancer death, right after lung cancer while surpassing breast, prostate, and colorectal tumors [4]. A potential explanation of this fact resides in the increased exposure of the general population to some of the multiple risk factors associated with the development of this cancer, which are, to a remarkable extent (66%), potentially modifiable [5], although non-modifiable and genetic risk factors should also be considered here [6]. Tobacco smoking, heavy alcohol drinking, a western dietary pattern (poor in vegetables and fruits), the presence of certain comorbidities (obesity, type 2 diabetes, and chronic pancreatitis), and deficient oral hygiene are some of the major risk factors more strongly associated with pancreatic cancer [7]. To the augmented incidence of this type of cancer, diagnostic difficulties—either in early screening or clinical manifestations—along with unsuccessful therapies and limited opportunities were added, as most of the patients were diagnosed with advanced metastatic disease [8,9,10]. Thus, emphasizing adequate preventative measurements andsearching for effective and novel approaches arise as critical points to improve the clinical management of pancreatic cancer, therefore limiting the negative impact of such a lethal disease.

In this context, advances in the field of molecular studies have fostered broader knowledge in the biological tumorigenesis of pancreatic cancer, also aiding in the elaboration of more accurate stratification and prognosis of patients with these tumors [11,12], in diagnosis and detection [13], as well as supporting the basis for novel therapeutical approximations [14]. In this sense, different molecules have been studied in pancreatic cancer, although further research is required to understand the role of these components and their possible uses in the clinical management of pancreatic cancer. Epidermal growth factor receptor (EGFR or ErbB1) is one of the best-characterized markers in pancreatic cancer. Prominently, the EGFR-KRAS pathway appears to provide a potential target in pancreatic cancer, leading to a plethora of altered cell processes and linking with the activation of additional molecular pathways [15]. Compelling evidence has proven the relevance of Wnt and its downstream effector β catenin in the malignization and therapeutic resistance of pancreatic cancers [16]. Integrated genomic analysis has reported multiple mutations affecting this pathway in pancreatic cancer [17]. Then, the aberrant activation of beta catenin induces the activation of critical oncogenes such as c-Myc or cyclin-D1, with important consequences in carcinogenic processes [18]. The last component, cyclin D1, is frequently dysregulated in many human cancers, acting as an allosteric modulator of cyclin-dependent kinase 4 and 6 (CDK4/CDK6) and regulating the transition of G1 to S phase [19]. ErbB2 (Also known as Her2/neu) is another member of the family of EGFR. Although its mutations are notably lower than EGFR, previous research has identified this component as a potential target to consider in some patients with pancreatic cancer [20]. Thus, the biological and translational applications in pancreatic cancer of all these elements place them as attractive molecular markers of study.

The aim of this article is to analyze the tissue expression of EGFR, Beta catenin, cyclin D1, CDK4, and ErbB2 in pancreatic cancer in order to find their association with the mortality and survival of patients with this tumor. Therefore, results are obtained in order to assess their usefulness as prognostic markers. 

## 2. Patients and Methods

### 2.1. Samples Collection

In our study, we used paraffin-embedded sections of pancreatic tissue obtained from 41 patients diagnosed with ductal adenocarcinoma who underwent surgery (curative resection of pancreatoduodenectomy), with patients being followed for 60 months. The diagnosis followed the principles of Esposito et al. [21] The present study was designed as an observational, analytical, and retrospective cohort study with longitudinal follow-up. The paraffin blocks and the different details with extensive clinical information about the patients and the follow-up data were retrospectively reviewed.

The study was carried out in accordance with the basic ethical principles of autonomy, beneficence, non-maleficence, and distributive justice, and its development followed the rules of Good Clinical Practice, the principles contained in the most recent Declaration of Helsinki (2013), and the Convention of Oviedo (1997). The data and information collected complied with the current legislation on data protection (Organic Law 3/2018 of December 5, Protection of Personal Data and Guarantee of Digital Rights and Regulation (EU) 2016/679).

### 2.2. Histopathological and Immunohistochemical Studies

Immunohistochemical studies were performed on paraffin-embedded pancreatic tissue samples. The antibody recovery step was described in the protocol’s specifications (Table 1). Antigen/antibody reactions were detected using the avidin-biotin (ABC) complex method, with avidin-peroxidase, following the protocols of Ortega et al. [22]. After incubation with the primary antibody (1 h 30 min), samples were incubated with a 3% BSA blocker (Catalog # 37525; Thermo Fisher Scientific, Inc., Waltham, MA, USA) and PBS overnight at 4 °C. The samples were then incubated with biotin-conjugated secondary antibody, diluted in PBS, for 90 min at room temperature (RT; Rabbit IgG (RG-96, 1:1000, Sigma-Aldrich/Mouse IgG (F2012/045K6072) 1:300, Sigma-Aldrich, St. Louis, MI, USA). The avidin-peroxidase conjugate ExtrAvidin^®^-Peroxidase (Sigma-Aldrich; Merck KGaA, Darmstadt, Germany) was used for 60 min at RT (1:200 dilution with PBS). Then, the level of protein expression was determined using a Chromogenic Diaminobenzidine (DAB) Substrate Kit (cat. no. SK-4100; Maravai LifeSciences, San Diego, CA, USA), which was prepared immediately before exposure (5 mL of distilled water, two drops of buffer, four drops of DAB, and two drops of hydrogen peroxide). The signal was developed with the chromogenic peroxidase substrate for 15 min at RT; this technique allows the detection of a brown stain. For the detection of each protein, sections of the same tissue were assigned as negative controls, substituting incubation with the primary antibody for a blocking solution (PBS). In all tissues, the contrast was performed with Carazzi hematoxylin for 15 min at RT.

### 2.3. Histopathological Assessment

Tissue sections were observed using a Zeiss Axiophot light microscope (Carl Zeiss, Oberkochen, Germany) equipped with an AxioCam HRc digital camera (Carl Zeiss, Oberkochen, Germany). Given the important role of the proteins studied, the histological evaluation was carried out according to the intensity of expression for immunohistochemical staining with Score. Therefore, histological samples from patients diagnosed with pancreatic cancer were classified as negative expression (0), low/medium (1), and high (3) using the IRS Score method [23]. For each established group of subjects, seven randomly selected microscopy fields were examined in each of the five sections. The individuals were classified as positive when the mean proportion of the labeled sample was superior or equal to 5% of the total sample. This was performed by calculating the total percentage of marked tissue in each microscopy field to obtain an average of the study sample, as previously described by Ortega et al. [22]. The observation and quantification of the samples were carried out independently by two researchers.

### 2.4. Statistical Analysis

First, a normality check of markers was carried out (Kolmogorov–Smirnoff, all *p* < 0.001). As we observed that they do not have a normal distribution, it was necessary to describe the results with medians and interquartile ranges by performing non-parametric tests. Mann–Whitney U test was used. To evaluate the association between clinicopathological and immunohistochemical parameters, a logarithmic rank test and Kaplan–Meier curves were developed for survival comparisons. In order to explore the correlation of the immunohistochemical parameters studied and the established prognosis of the variables, a univariate analysis and a Cox proportional hazards regression analysis were used. All statistical analyses were performed using SPSS 22.0 software (SPSS Inc., Chicago, IL, USA). Values of *p* < 0.05 were considered significant.

## 3. Results

### 3.1. Clinical and Sociodemographic Characteristics

The present study was designed as an observational, analytical, retrospective cohort study with longitudinal follow-up. A total of 41 patients were analyzed, with a median age of 72.00 (45.00–88.00) years, of which 65.85% were men (*n* = 27) and 34.15% were women (*n* = 14). The clinical and sociodemographic characteristics are collected in Table A1. Tumor Stage is collected in Supplementary Table S1. In global terms, the survival of patients with a diagnosis of pancreatic cancer was 8.00 (2.98–13.02) months. 

### 3.2. Patients with Higher Expression of CDK4, Cyclin-D1, B-Catenin, and EGFR Show Lower Survival to Pancreatic Cancer

**Figure 1:**
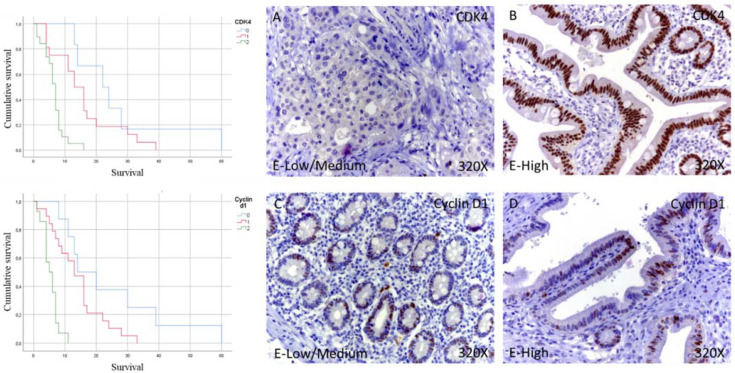
Kaplan–Meier curves for survival time according to tumor expression (**left**) and images showing the protein expression of CDK4 and Cyclin-D1 in patients diagnosed with pancreatic cancer (**right A**–**D**). E-Low/Medium = tissue expression classified as low/medium; E-High = tissue expression classified as high; 320× magnification. Histological samples from patients diagnosed with pancreatic cancer were classified as negative expression (0), low/medium (1), and high (3) using the IRS Score method *n* = 41.

In the case of Cyclin-D1, immunohistochemical studies showed that 19.51% of patients with pancreatic cancer did not show tissue expression for this component, while in the case of patients with low/medium expression, it was 46.34%. In contrast, 34.15% showed high levels of Cyclin-D1. In total, 80.49% of the patients showed expressions of Cyclin-D1 (Table 2 and Figure 1C,D).

**Figure 2:**
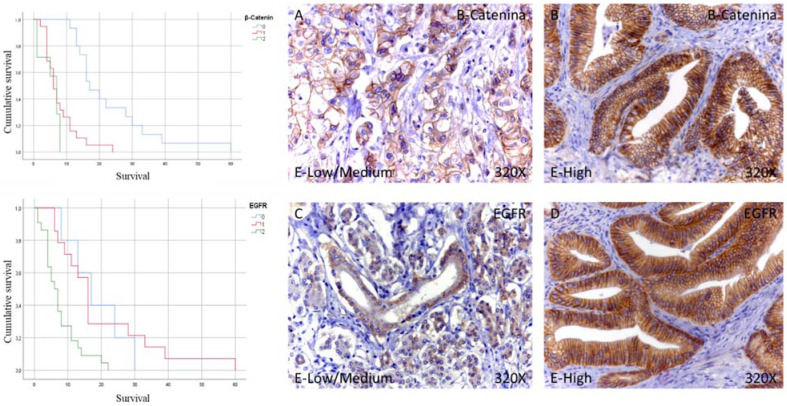
Kaplan–Meier curves for survival time according to tumor expression (**left**) and images showing the protein expression of beta catenin and EGFR in patients diagnosed with pancreatic cancer (**right A**–**D**). E-Low/Medium = tissue expression classified as low/medium; E-High = tissue expression classified as high; 320× magnification. Histological samples from patients diagnosed with pancreatic cancer were classified as negative expression (0), low/medium (1), and high (3) using the IRS Score method *n* = 41.

The median survival months for patients with pancreatic cancer and negative for the tissue expression of Cyclin-D1 was 14.00 (4.29–23.70) months. However, in the case of patients with low-medium expression, it was 13.00 (9.44–16.55) months, falling to 5.00 (2.55–7.44) months in patients with high expression (Figure 2B). Global comparisons showed that the significance value was *p* < 0.001, with its Hazard Ratio (HR) of 12.07 (3.76–38.67) in patients with high expressions of Cyclin-D1.

Next, immunohistochemical studies showed that 17.08% of patients with pancreatic cancer did not show tissue expression for B-catenin, while in the case of patients with low/medium expression, it was 46.34%. In contrast, 36.58% showed high levels of B-catenin. In total, 82.92% of the patients showed B-catenin expression (Table 2 and Figure 2A,B).

The median survival months for patients with pancreatic cancer and negative for tissue expression of B-catenin was 17.00 (12.46–21.54) months. However, in the case of patients with low-medium expression, it was 6.00 (4.29–7.71) months, falling to 7.00 (4.66–9.34) months in the case of patients with high expression (Figure 2). Global comparisons showed that the significance value was *p* < 0.001, with its Hazard Ratio (HR) being 10.62 (3.42–32.94) in patients with high B-catenin expression.

In the case of EGFR, immunohistochemical studies showed that 12.19% of patients with pancreatic cancer did not show tissue expression of the tumor for EGFR, while in the case of patients with low/medium expression, it was 34.15%. In contrast, 53.66% showed high EGFR levels. In total, 87.81% of the patients showed EGFR expression (Table 2 and Figure 2C,D).

Median survival months for EGFR tissue-negative pancreatic cancer patients was 17.00 (8.41–25.59) months. However, in the case of patients with low-medium expression, it was 16.00 (13.51–18.48) months, decreasing to 6.00 (3.70–8.29) months in the case of patients with high expression (Figure 2). Global comparisons showed that the significance value was *p* < 0.001, with its Hazard Ratio (HR) of 3.26 (1.18–9.02) in patients with high EGFR expression.

### 3.3. ErbB2 Is a Central Marker Associated with Mortality

**Figure 3:**
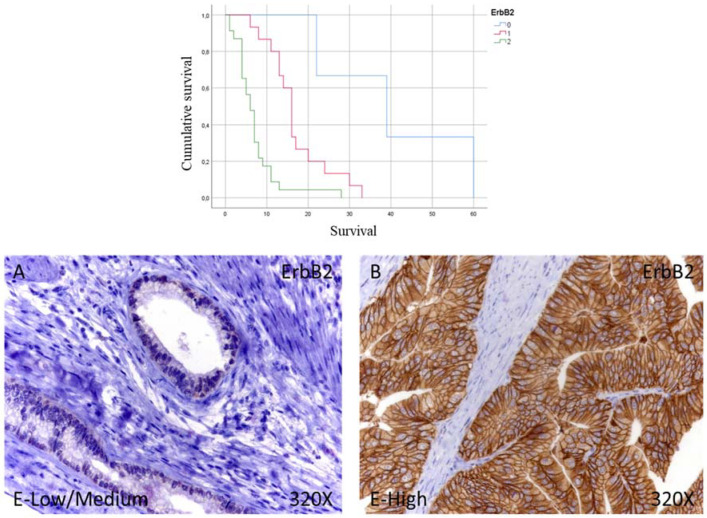
Kaplan–Meier curves for survival time according to tumor expression (**top**) and images showing the protein expression of Erb2 in patients diagnosed with pancreatic cancer (**bottom A**,**B**). E-Low/Medium = tissue expression classified as low/medium; E-High = tissue expression classified as high; 320× magnification. Histological samples from patients diagnosed with pancreatic cancer were classified as negative expression (0), low/medium (1), and high (3) using the IRS-Score method *n* = 41.

Immunohistochemical studies showed that 7.33% of patients with pancreatic cancer did not show tissue expression for ErbB2, while 36.58% correspond to patients with low/medium expression it was. In contrast, 56.09% showed high levels of ErbB2. In total, 92.67% of the patients showed expression of ErbB2 (Figure 3A,B).

The median survival months for patients with pancreatic cancer and negative for tissue expression of ErbB2 was 39.00 (11.79–66.21) months; however, in the case of patients with low-medium expression, it was 16.00 (14.21–17.79) months, falling to 6.00 (4.43–7.56) months for patients with high expression (Figure 3). Global comparisons showed that the significance value was *p* < 0.001, with its Hazard Ratio (HR) being 27,280 (3.33–223.52) in patients with high ErbB2 expression.

## 4. Discussion

Molecular studies have aided in the understanding of pancreatic cancer tumorigenesis, also supporting the clinical management of such a deadly malignancy. Our study has provided a direct association between pancreatic cancer survival and the overexpression of EGFR, beta-catenin, cyclin D1, CDK4, and ErbB2. Thus, the detection of these molecules in the tissue could serve as indicators of poor prognosis in the affected patients.

ErbB2 is the marker more clearly related to the clinical outcome in our study. Although ErbB2 is commonly described in breast tumors [24], a growing number of studies are starting to highlight the valuable role of this molecule as a biomarker in non-breast cancers. However, the usefulness of ErbB2 in the prognosis of pancreatic cancer is still controversial. Biologically, this molecule appears to be implicated in the progression and malignancy of pancreatic cancer, collaborating with additional signaling molecules [25,26,27]. The protein expression of ErbB2 is upregulated between 7 to 61% of patients with pancreatic cancer and about 2 to 24% of patients show ErbB2 gene amplification [28]. Nonetheless, previous studies demonstrated that ErbB2 exerts its oncogenic effect when it is overexpressed and not only when it is amplificated [29], thereby supporting the value of studying this component in the tissue and not only from a genetic perspective. On the one hand, some researchers did not find any associations between ErbB2 overexpression and in clinical outcomes in patients with pancreatic ductal adenocarcinoma [30,31,32]. Conversely, [33] claimed the relevance of ErbB2 as an independent prognostic factor in patients with pancreatic cancer, associated with poorer outcomes. In the same line, a recent work conducted by [34,35] deepens the prognostic value of ErbB2 in pancreatic cancer. They show that approximately 42% of patients show tissue expression of this protein, with significant clinical consequences for these individuals. In addition, not only those with high ErbB2 expression but also patients with low or no expression of this component could be associated with poor outcomes and reduced survival, due to the ErbB2 genetic heterogeneity identified in approximately in the 70% of patients studied. Hence, it could be concluded that either protein expression or gene heterogeneity involving ErbB2 is a significant prognostic marker in pancreatic cancer. Our results support the role of protein expression of this component in establishing survival and anticipate clinical outcomes in patients with pancreatic tumors. Notwithstanding, the role of ErbB2 in pancreatic cancer prognosis is well defined, and its therapeutical implications are not as promising as expected. In this line, a phase II multicenter study evaluated the toxicity and efficacy of combined trastuzumab plus capecitabine in patients with ErbB2 overexpressed, and it did not observe any significant improvement in this therapy versus standard chemotherapy [36], showing the clinical difficulties of targeting this component. 

EGFR is another ErbB receptor implicated in the development of multiple malignancies [37]. EGFR is overexpressed in 30 to 95% according to previous studies, where its expression correlated with advanced stages of the disease and the presence of metastasis [38]. However, similarly to ErbB2, its clinical significance in the survival of patients with this cancer is contradictory. Thus, some argue that EGFR is overexpressed in pancreatic cancers, independently from histopathological features without predicting survival [39]. Other studies added that, rather than a marker of worse prognosis, EGFR is associated with positive outcomes when coexpressed with one of its ligands: the epidermal growth factor (EGF) [40]. Otherwise, some studies that observed a direct association between the cytoplasmic expression of EGFR and shorter survival in comparison to patients with lower expression [41,42] discussed that the higher expressions of both EGFR and CXCR4 are associated with poor prognosis, as they might share some common synergic mechanisms. In this line, our study shows that the combined use of all these markers might altogether be implicated in the reduced survival observed in patients overexpressing these molecules. 

Beta-catenin and one of its main downstream effectors, cyclin D1, are also augmented in our study, being strongly correlated with a poorer prognosis in patients with pancreatic cancer. Beta-catenin is activated by the Wnt signaling pathway, playing multiple roles in pancreatic cancer biology. In this line, an altered expression of this component appears to be associated with greater invasiveness [43] with the inflammatory environment [44], drug resistance [16], and aberrant molecular pathways [45]. Because of that, we observed a reduced survival in patients overexpressing this component. Our study is consistent with previous research, where the nuclear overexpression of this component was associated with poor clinical outcomes in patients with pancreatic cancer [46]. Furthermore, Qiao et al. [47] demonstrated that increased cytoplasmic levels of beta-catenin and reduced membrane detection were both linked with cyclin D1 overexpression and poor prognosis in pancreatic cancer. The increased detection of cyclin D1 could also serve as a predictor marker of poor prognosis in patients who undergo a surgery procedure [48]. Interestingly, Bachmann et al. [49] reported different survival rates according to the polymorphic variants of the cyclin D1 gene (CCND1), finding that patients with the G870A variant were associated with the increased protein expression of cyclin D1 and worse clinical outcomes. Frequently, cyclin D1 forms a molecular complex with CDK4, another marker significantly increased in our study. Previous research has described the role of cyclin D1 and CDK4 in augmented cell proliferation and poor prognosis in some types of cancer [50]. A wide variety of oncogenic targets are regulated by this complex, and both elements are frequently altered in pancreatic tumors, making them a profitable therapeutic approach for future studies [19,51,52]. Although there are few studies evaluating the role of CDK4 as a prognostic biomarker, its overexpression is associated with carcinogenic processes, regulating inflammatory cytokine signaling and inducing a metastatic phenotype and cell growth [53]. Hence, the increased detection of CDK4 could be associated with a malignant switch of pancreatic cells, acting together with cyclin D1 and altering diverse molecular routes. 

## 5. Conclusions

Our study has evidenced the prognostic role of a plethora of molecular markers, including ErbB2, EGFR, beta-catenin, cyclin D1, and CDK4. Of them, we would like to highlight the clinical significance of ErbB2 in the survival rates of patients overexpressing this component. For all these reasons, the study of these molecules allows us to demonstrate the importance of their implementation in the histological studies carried out in these patients. All this can allow us to direct efforts and effectiveness in these patients for a comprehensive and personalized diagnosis and treatment.

## Figures and Tables

**Table 1:** Primary antibodies used, together with the dilutions and those specified of the protocol.

Antígen	Dilution	Provider	Protocol Specifications
CDK4	1:250	Vitro, MAD-000597QD-3/V	-
Cyclin D1	1:500	Vitro, MAD-000630QD-3/V	Preincubation with Tris-EDTA Buffer pH9 and incubation with 0.1% TTX (Triton × 100 in TBS) for 5 min
Beta Catenin-1	1:250	Vitro, MAD-000699QD-3/V	Preincubation with Tris-EDTA Buffer pH9 and incubation with 0.1% TTX (Triton × 100 in TBS) for 5 min
EGFR	1:300	Vitro, MAD-000664QD-3/V	-
ErbB2	1:500	Vitro, MAD-000308QD-3/V	-

**Table 2:** Percentage of positive expression for CDK4, Cyclin-D1, B-catenin, and EGFR in pancreatic cancer, classified according to tissue expression levels. E-Negative = negative expression; E-Low/Medium = tissue expression classified as low/medium; E-High = tissue expression classified as high.

	CDK4 *n* (Ratio%)	Cyclin D1 *n* (Ratio%)	B-Catenin *n* (Ratio%)	EGFR *n* (Ratio%)
E-Negative	6 (14.63)	8 (19.51)	7 (17.08)	5 (12.19)
E-Low/medium	16 (39.02)	19 (46.34)	19 (46.34)	14 (34.15)
E-High	19 (46.35)	14 (34.15)	15 (36.58)	22 (53.66)

## Data Availability

The datasets used and/or analyzed during the present study are available from the corresponding author upon reasonable request.

## Supplementary Materials

The following supporting information can be downloaded at https://www.mdpi.com/article/10.3390/curroncol29040198/s1, Table S1: Clinical and labeling data of each protein.

## Appendix A

**Table A1:** Clinical and sociodemographic characteristics of patients diagnosed with pancreatic cancer included in the study. IQR = Interquartile range; *n* = number of patients.

Age (Median (IQR))	72.00 (45.00–88.00)
Sex (*n* (Ratio%))	
Men	27 (65.85)
Women	14 (34.15)
Smoking	18 (43.90)
Drinking	11 (26.83)
Obesity	2 (4.88)
Type II diabetes	15 (36.58)
Chronic pathologies	4 (9.76)
Prior malignant neoplasms	11 (26.83)
