# Supplementary material for: Implication of ERBB2 as a Predictive Tool for Survival in Patients with Pancreatic Cancer in Histological Studies
Source: Curr Oncol. 2022 Mar 30;29(4):2442–53. doi: 10.3390/curroncol29040198 (PMC9027548; doi:10.3390/curroncol29040198)
Supplement: Supplementary file 1 [file curroncol-29-00198-s001.zip › curroncol-1624224-supplementary.pdf]

Supplementary Materials

Table S1. Clinical and labeling data of each protein.

nº	ESTADIO	EDAD	SEXO (1 h, 2 m)	TUMORES (mama, ovario, melan, colon)	Ca 19.9 U/mL (0–37)	CEA ng/mL (0–5)	AFP ng/mL (0–13.4)	CDK4	Cyclin d1	β-Catenin	EGFR	ErbB2	SUPERVIVENCIA EN MESES
1	<IV	67	1	1	42.36	27	7	2	1	2	2	2	7
2	IV	82	1	1	44.91	34	6.01	2	2	1	2	2	8
3	IV	61	2	0	69.52	2.2	3.8	0	0	0	2	1	14
4	<IV	62	2	0	102	2.6	1.31	1	0	0	2	2	13
5	<IV	70	1	0	189.6	5.74	1.73	2	0	0	2	2	11
6	<IV	45	1	0	1079	22.68	2.4	2	2	1	2	1	6
7	IV	63	1	0	178	13.84	4.39	2	2	1	2	2	7
8	<IV	77	1	0	86	3.79	1.93	0	1	0	1	2	28
9	IV	71	2	0	2.62	23.56	1.07	2	1	2	2	1	8
10	<IV	57	2	0	1.000	5.62	5.77	2	1	2	2	2	1
11	<IV	76	1	1	635.4	11.31	1.98	1	2	1	2	2	11
12	<IV	77	1	1	8.4	2.23	1.12	2	2	2	2	2	1
13	<IV	72	1	0	1.08	2.71	1.12	0	1	1	0	1	13
14	<IV	73	2	0	2.933	3.26	16.13	2	0	2	0	2	8
15	IV	66	1	0	340.6	4.9	3.39	0	1	1	0	1	24
16	<IV	72	2	1	102.1	2.42	2.66	2	1	1	2	2	5
17	<IV	83	1	0	805	17	5.4	1	1	1	2	2	4
18	<IV	73	2	0	29.31	1.54	7.62	1	0	0	2	1	20
19	<IV	69	1	1	3.47	10.24	2.24	0	0	0	1	0	60

20	<IV	79	2	0	2324	12.6	5.46	1	1	1	1	1	11
21	IV	72	1	0	721.6	1.8	1.09	1	1	0	1	1	13
22	<IV	88	1	1	0.6	1.82	1.24	2	1	1	1	2	9
23	IV	64	2	0	45.25	3.89	4.33	1	1	0	1	1	16
24	IV	57	1	1	47.42	8.6	6.48	1	1	0	1	1	16
25	<IV	61	2	0	203	4.19	2.1	2	2	1	2	2	4
26	IV	74	1	0	8868	8.05	1.22	1	2	1	2	2	4
27	<IV	70	1	0	56.01	5.43	0.98	1	1	0	1	1	33
28	IV	74	2	0	10.000	57.5	30.79	2	1	1	1	2	6
29	<IV	67	1	0	160.9	7.92	1.72	1	0	0	0	1	30
30	<IV	67	2	0	87.67	7.84	1.56	1	0	0	1	0	39
31	<IV	74	1	0	152.6	1.98	0.61	2	1	1	1	1	16
32	<IV	75	1	0	59.16	1.56	2.66	0	1	0	2	0	22
33	<IV	69	1	0	4.64	4.24	2.32	1	1	0	1	1	16
34	<IV	83	1	0	10.000	498	2.87	2	2	1	2	2	2
35	<IV	85	1	1	114.8	1.78	1.86	2	2	1	2	2	7
36	IV	82	1	0	1.000	40.46	1.39	1	2	2	2	2	5
37	<IV	73	1	0	31.03	2.77	1.51	2	2	2	1	2	7
38	<IV	67	2	0	48.32	7.85	7.25	1	1	0	0	1	17
39	IV	76	1	1	2145	6.52	2.56	2	2	1	1	2	6
40	IV	77	2	0	1774	4.58	3	2	2	1	2	2	4
41	<IV	67	1	1	36.28	3.44	1.46	1	2	1	2	2	4
